# Differential Psychological and Social Impact of the COVID-19 Pandemic on Spanish Youth With and Without Non-Suicidal Self-Injury: A Longitudinal Analysis

**DOI:** 10.62641/aep.v54i2.2043

**Published:** 2026-04-15

**Authors:** Carlos Schmidt, Daniela Otero, Juan C. Pascual, Soledad Romero, Joaquim Puntí, Anaís Lara, Anna Sintes, Iria Méndez, Stella Nicolaou, Joaquim Soler, Daniel Vega

**Affiliations:** ^1^Department of Psychiatry, Hospital Universitari d’Igualada (Consorci Sanitari de l’Anoia), Fundació Sanitària d’Igualada, 08700 Barcelona, Spain; ^2^Department of Psychiatry, Hospital de la Santa Creu i Sant Pau, Institut d’Investigació Biomèdica-Sant Pau (IIB-SANT PAU), 08041 Barcelona, Spain; ^3^Universitat Autònoma de Barcelona (UAB), 08193 Barcelona, Spain; ^4^Mental Health and Psychiatry Department, Consorci Hospitalari de Vic. Institut de Recerca i Innovació en Ciències de la Vida i de la Salut a la Catalunya Central (IRIS-CC) 08500, Barcelona, Spain; ^5^Centro de Investigación Biomédica en Red de Salud Mental, Instituto de Salud Carlos III, 28029 Madrid, Spain; ^6^Instituto de Neurociencias, Hospital Clínic de Barcelona, IDIBAPS, 08036 Barcelona, Spain; ^7^Salud Mental, Corporació Sanitària Parc Taulí de Sabadell, 08208, Barcelona, Spain; ^8^Servicio de Psiquiatría, ALTHAIA, Xarxa Assistencial de Manresa, 08243, Barcelona, Spain; ^9^Servicio de Psiquiatría y Psicología, Hospital de Sant Joan de Déu, Esplugues de Ll., 08950 Barcelona, Spain; ^10^Servei de Psiquiatria & Psicol Infantil Juvenil. Hospital Universitari Mutua Terrassa, Fundació Recerca Mutua Terrassa, 08221 Barcelona, Spain

**Keywords:** non-suicidal self-injury, COVID-19, self-harm, young adult, perceived social support

## Abstract

**Background:**

Non-suicidal self-injury (NSSI) in adolescents and young adults is a serious public health concern. The COVID-19 pandemic significantly impacted mental health worldwide. This longitudinal study aimed to investigate the differential impacts of COVID-19 on psychological health, social support, and academic performance among young adults with and without previous history of NSSI.

**Methods:**

From an initial sample of 603 college students, 241 (40%) completed this 2.5-year follow-up study. The first assessment was in January-February/2020 (pre-pandemic) and the second in June-July/2022 (post-pandemic). Participants were grouped based on the presence or absence of NSSI at baseline. Variables assessed included sociodemographic data, academic performance, COVID-19-related experiences, clinical characteristics, and perceived social support.

**Results:**

A significant reduction in the prevalence of NSSI behaviors was observed over the follow-up period, decreasing from 35% to 8.7%. The NSSI group endorsed worse academic performance post-pandemic. While they maintained stable clinical severity with no observed worsening, during pandemic period they experienced an improvement in perceived social support. In contrast, the Non-NSSI group experienced a decline in perceived social support during the same period.

**Conclusions:**

Contrary to previous studies, our findings indicate that young adults with NSSI significantly reduced self-harm behaviors after the COVID-19 pandemic. Although their academic performance was negatively affected, their clinical severity and social support did not worsen compared to those without NSSI. Findings indicate that the COVID-19 outbreak did not increase NSSI behaviors or exacerbate psychopathology in individuals with NSSI.

## Background

Non-suicidal self-injury (NSSI) represents a critical issue in mental health that has increasingly gained scientific and societal attention as a serious public health concern. NSSI is defined as the intentional, direct infliction of harm upon one’s body tissue in the absence of suicidal motivation and commonly includes behaviors such as cutting, scratching, and pinching [1, 2]. Emerging predominantly during early adolescence or early adulthood, NSSI exhibits prevalence rates of 17.2% among adolescents and 13.4% among young adults in the community [3, 4]. Recurrent NSSI has shown increased prevalence, bearing potential negative consequences such as diminished interpersonal, academic, and daily functioning, compromised mental health, heightened risk of future suicide attempts, and accidental death [2]. A systematic review of longitudinal studies indicated that NSSI prevalence rates peak in middle adolescence and decline toward the end of adolescence or young adulthood (18 years) [5].

The onset of the Coronavirus Disease 2019 (COVID-19) has posed the most significant health and socio-historical crisis of the 21st century, threatening global health, economic stability, and social systems [6]. The initial outbreak precipitated an increase in cases and deaths, prompting the implementation of urgent measures to stop the virus’s spread, including the closure of schools and the paralysis of all non-essential activities. Forced quarantine, social distancing, self-isolation, mobility restrictions, and the limitation of leisure and sports activities have characterized interpersonal, occupational, and social interactions since the pandemic’s onset. These measures have notably affected young people [7, 8]. Previous evidence shows that the COVID-19 pandemic seriously affected the perceived social support (PSS) and this impacted on quality of life and mental health [9, 10]. PSS refers to the feeling of being valued and supported by one’s social network and has been considered an important protective factor against the urge to self-harm in young people [9, 10, 11, 12]. Critically, youth with a history of NSSI often report a diminished perception of social support [12, 13]. This perceived lack of support may motivate self-injury as a way of coping with interpersonal difficulties [14]. In contrast, social support facilitates emotional self-regulation and effective coping with difficult emotions, social and interpersonal challenges, particularly among young people [15, 16]. These unpredictable and disruptive stressors caused by the pandemic have precipitated mental distress and psychiatric disorders. The COVID-19 crisis constitutes a socio-historical event with considerable implications for mental health. Furthermore, throughout this period, youth have endured repeated disruptions to schooling, social interactions, peer relationships, and extracurricular activities throughout the pandemic [7]. Notably, during the course of the COVID-19 pandemic, there has been an exacerbation of severe mental distress among children and adolescents, increasing youth mental health disorders such as depression, anxiety, and eating disorders [17, 18, 19]. Moreover, compelling evidence indicates increases in suicidal ideation, suicide attempts, and self-harm during the pandemic. Specifically, a meta-analysis examining 11.1 million adolescent emergency department visits across 18 countries between January 2020 and December 2022 found an increase in suicide attempt (rate ratio [RR] 1.22, 90% *CI* 1.08–1.37). However, the increase in self-harm was more modest (RR 1.18, 90% *CI* 1.00–1.39) and limited to older adolescents aged 16–17 years [20].

Regarding suicidal behavior, research conducted during the pandemic has yielded discrepant findings depending on the observation period’s duration and its relationship with the COVID-19 first wave [21]. Initial confinement periods with short follow-up durations failed to discern significant changes in suicide rates compared to previous periods, with some studies even suggesting a decrease in psychiatric emergency department visits for suicidal ideation or attempts [21, 22, 23]. Conversely, studies with prolonged follow-up periods have reported an increase in youth seeking urgent psychiatric care due to suicidal ideation or attempts [20, 21]. Extending the observation period into the last period of the COVID-19 pandemic (year 2021) revealed a distressing increase in psychiatric emergency department visits among children and adolescents attributable to suicidal ideation or attempts [20, 21, 24]. Moreover, recent research suggests that the estimated prevalence of NSSI in adolescents remained similar before and after COVID-19 [25].

Therefore, the COVID-19 pandemic represents a serious adverse event with considerable implications for youth mental health, particularly concerning suicidal ideation, attempts, and self-harm among adolescents. However, the differential impact of the pandemic on adolescents with and without prior suicide acts or NSSI remains underexplored, largely due to a reliance on cross-sectional data collected after the pandemic began. The present study addresses this gap by longitudinally exploring the effect of the pandemic on mental health (i.e., emotion dysregulation, psychological distress) and social support in college students with and without a history of NSSI. Specifically, we are interested in exploring two key questions: (i) whether the pandemic increased the frequency of self-injury in youth with a history of NSSI; and (ii) whether the effect of the pandemic on mental health and social support was greater in those youth with a history of NSSI.

We hypothesize that the pandemic had a more pronounced impact, increasing clinical severity and decreasing perceived social support (PSS) among young individuals with a previous history of NSSI. Leveraging a prospective follow-up study initiated before the pandemic, we have a unique opportunity to investigate patterns of change in self-injurious behaviors among youth with and without a previous history of NSSI during this period.

## Methods

### Participants

A total of 603 college students (*M*_𝑎𝑔𝑒_ = 20.42, *SD* = 1.69; 81.6% female) were recruited from Autonomous University in Spain between January-February 2020, one month prior to the onset of COVID-19 pandemic (T1). Subsequently, participants were re-contacted via email between June-July 2022, after the pandemic had ended (T2), to participate in a follow-up assessment. Finally, 241 participants (40% of the original sample) responded to the follow-up survey (*M*_𝑎𝑔𝑒_ T2 = 22.41, *SD* = 1.79; 83.8% female).

These participants formed the final sample of study. The inclusion criterion for the non-NSSI group was having no history of NSSI or engaging in self-injurious behaviors in the past year. For the NSSI group, the inclusion criterion was the presence of ≥5 days of NSSI behaviors in the last year. The exclusion criterion for both groups was the inability to understand or read Spanish*.*

### Procedure

The present study was previously planned and did not consider the onset of COVID-19, the health and social crisis, or the quarantine in its development. However, it provided an excellent opportunity to study the pandemic’s effects on adolescents and young individuals with NSSI.

The sample of college students was recruited via an online survey to assess NSSI behaviors, clinical variables and social support. Participants were invited to participate via email, informed that participation was voluntary, and provided with information about the purpose of the study. In addition, they were informed of the possibility of receiving monetary compensation (€30) as recognition for their time and effort. Interested participants gave their informed consent electronically and were then redirected to the online survey, which consisted of four different sections: (i) sociodemographic information; (ii) information on NSSI behaviors (i.e., frequency, methods, age of onset, and functions); (iii) clinical variables; and (iv) measures of perceived social support. The survey took approximately 30 to 40 minutes to complete.

The same methodology was used in the 2.5-year follow-up to obtain longitudinal data. Because the follow-up coincided with post-pandemic period, additional questions were included about: (i) information on COVID-19 and its consequences (e.g., pandemic quarantine) and (ii) the pandemic’s effect on academic performance and mental health. All procedures were approved by the Clinical Research Ethics Committee of Bellvitge University Hospital (protocol number: PR330/17, 25/1/2018). The study was conducted in accordance with the Declaration of Helsinki.

### Instruments

#### Non-Suicidal Self-Injury Disorder Scale (NSSIDS)

This self-report measure assesses the criteria for Non-Suicidal Self-Injury Disorder proposed in Diagnostic and Statistical Manual of Mental Disorders (DSM-5) [26]. The NSSIDS has a total of 20 items. Participants reported the number of days in the past year in which they engaged in NSSI (criterion A – recent), and whether or not they had engaged in NSSI for 5 or more days within a year prior to the last year (criterion A – past), using a frequency count item. Criterion B assesses the functions of NSSI using three items, each rated on a 7-point Likert scale ranging from 1 (Never) to 7 (Always): (i) to relieve negative feelings or thoughts (i.e., affect regulation); (ii) to cope with interpersonal problems (i.e., interpersonal regulation); and (iii) to create a positive feeling. In the current study, criterion A (recent) was used as a continuous measure of recent engagement in NSSI (i.e., 5 or more days of NSSI in the past year), and criterion B was used as a measure of NSSI-function. The NSSIDS presented good internal reliability (Cronbach’s α = 0.88) in the original version [26]. In the present study, the internal reliability was acceptable at Time 1 (α = 0.77) and Time 2 (α = 0.76).

#### Inventory of Statements about Self-Injury (ISAS)

This self-report questionnaire assesses NSSI frequency, methods and age of onset [27]. The ISAS is comprised of 36 items divided into two main sections. The Section I of ISAS evaluates the frequency of 12 NSSI behaviors: hitting self, biting, burning, carving, cutting, wound picking, needle-sticking, pinching, hair pulling, rubbing skin against rough surfaces, severe scratching, and swallowing chemicals. Section II measures 13 NSSI functions. This section was not used in the present study. The purpose of utilizing the ISAS was to characterize NSSI behavior (e.g., methods). The original ISAS showed good internal reliability (α = 0.84) [27]. This study had an acceptable internal reliability at Time 1 (α = 0.75) and Time 2 (α = 0.74). The Spanish version of the ISAS was administered [28].

#### The Borderline Personality Questionnaire (BPQ)

This self-report questionnaire assesses BPD traits or symptoms [29]. BPQ is an 80-item self-report measure that assesses BPD traits and symptoms across nine subscales, utilizing a dichotomous (True/False) response format based on the DSM-IV criteria: Impulsiveness, Affective Instability, Abandonment, Relationship, Self-Image, Suicide/Self-Mutilation, Emptiness, Intense Anger, and Quasi-Psychotic States. The BPQ total score was used as a continuous measure of general BPD traits. Higher scores indicate a greater presence of borderline traits. The Spanish version of the BPQ presents moderate to high internal reliability (Cronbach’s α = 0.78 to 0.93) across the nine scales [29]. In the current study, the BPQ total score showed good internal reliability at Time 1 (α = 0.84) and Time 2 (α = 0.85).

#### Brief Version of the Difficulties in Emotion Regulation Scale (DERS-18) 

The Spanish translation of this scale was used. This 18-item self-report assesses emotion dysregulation through six subscales using a 5-point Likert scale ranging from 1 (Almost Never) to 5 (Almost Always) [30]: (i) lack of emotional awareness, (ii) lack of emotional clarity, (iii) inability to engage in goal-directed behavior when feeling emotional, (iv) engagement in impulsive behavior when feeling emotional, (v) nonacceptance of emotions, and (vi) inability to access emotion regulation strategies. The total DERS score was used as a measure of emotion dysregulation. Higher scores on the scale indicate greater emotional dysregulation. The total DERS score presents a high internal reliability (Cronbach’s α = 0.91) [30]. In the present study, the internal reliability was good at both Time 1 (α = 0.86) and Time 2 (α = 0.85).

#### Depression Anxiety Stress Scales (DASS-21)

This self-report assesses current psychological distress during the previous week based on the frequency of 21 emotional symptoms across three subscales: Depression, Anxiety, and Stress [31]. All items are rated on a 4-point frequency/severity scale, ranging from 0 (Did not apply to me at all) to 3 (Applied to me very much, or most of the time). Higher scores indicate greater severity. The Spanish version of the DASS-21 showed moderate to good internal reliability (Cronbach’s α = 0.84, 0.70, and 0.82 for each of the subscales, respectively) [31]. In the present study, the internal reliability was excellent at both Time 1 (α = 0.93) and Time 2 (α = 0.92).

#### Multidimensional Scale of Perceived Social Support (MSPSS)

The Spanish version of the MSPSS (https://www.heardalliance.org) was used [11]. This is one of the most broadly used scales to rate social support. The MSPSS is a 12-item measure rated on a 7-point Likert scale ranging from 1 (Very Strongly Disagree) to 7 (Very Strongly Agree). It assesses three areas of social support derived from: (i) family, (ii) friends, and (iii) significant others. Higher scores indicate higher PSS. The Spanish version of the MSPSS showed good internal reliability (α = 0.88) [32]. In the current study, the internal reliability was good at both Time 1 and Time 2 (α = 0.90).

### Statistical Analyses

Analyses were conducted using data from participants who had complete measurements at both T1 and T2 (n = 241). First, attrition analyses were performed by comparing responders vs. non-responders at T2 using the Student’s *t*-test for continuous and Pearson’s Chi-square test (χ^2^) for categorical variables. This was done to determine the reliability of the results and ensure the final sample included in the analyses was representative of the original sample.

Second, participants were grouped based on the presence of ≥5 days with NSSI behaviors in the last year (NSSI group; n = 85) or the absence (Non-NSSI group; n = 156) of a history of self-injury at T1. Between-group differences for sociodemographic, COVID-19 pandemic variables, academic status, clinical measures, and PSS at T2 were examined. For these analyses, Student’s *t*-test was used for continuous variables when the assumption of normality was met, and the nonparametric Mann-Whitney U test was used when normality was not met. Pearson’s Chi-square test was used for categorical variables.

Third, McNemar tests were used to assess changes in NSSI frequency from T1 to T2. This test was used to compare the pre-post change in the distribution of proportions in dichotomous variables.

Fourth, linear mixed models (LMMs) were performed using a restricted maximum likelihood estimation method (REML) to assess whether groups at T1 (i.e., NSSI vs. Non-NSSI group) showed changes in clinical measures and PSS at follow-up (T2). For these analyses, Group (i.e., NSSI vs. No NSSI), Time of clinical measures and PSS (i.e., T1 vs. T2), and their interaction (Group × Time) were modeled as fixed effects. The intercept for participants was defined as a random effect. LMM analyses allow for modeling the temporal dynamics of clinical measures and PSS from T1 to T2 (i.e., Time effects) and for examining the differences between the NSSI Groups (i.e., Group effects) based on NSSI status at T1. Prior to model interpretation, the normality assumption of both the conditional residuals and the random effects was evaluated using the Shapiro-Wilk test and visual inspection of the Q-Q plot and the histogram of the residuals. LMM analyses were conducted using the lme4 package in RStudio (version 4.5.1, R Foundation for Statistical Computing, Vienna, Austria).

Fifth, in order to evaluate which factors are associated with NSSI persistence, logistic regression analyses were conducted to determine whether clinical status and PSS at T1 predicted the maintenance of NSSI behavior at T2. The analysis used the presence versus absence of NSSI at T2 (NSSI vs. No NSSI) as the dichotomous outcome. Predictors included age and gender as potential covariates, along with the clinical and PSS measures. In the first step, univariate logistic regression analyses were conducted. In a second step, predictors that showed a statistically significant effect in the univariate analyses were then entered into a multivariate logistic regression analysis using a stepwise procedure. Logistic regression analyses yielded odds ratios (*ORs*) presented with 95% Confidence Intervals (95% *CIs*). Descriptive analyses were performed using IBM SPSS Statistics version 24.0 (IBM Corp., Armonk, NY, USA) and statistical tests were performed using RStudio.

## Results

### Sociodemographic and Clinical Baseline Characteristics.

From the overall sample of 603 college students, 241 (40.0%) completed both T1 and T2 assessments, forming the final sample for the follow-up study. Differences between responders vs. non-responders were assessed, and no significant differences were found in age (*t* = 0.22, *p* = 0.82, *d* = 0.01), sex (χ^2^ = 0.87, *p* = 0.34), academic performance (χ^2^ = 1.74, *p* = 0.41), or social and clinical measures (MSPSS: *t* = –1.03, *p* = 0.30, *d* = 0.001; BPQ Total:* t* = 1.26, *p* = 0.20, *d* = 0.01; DERS-18: *t* = 0.33, *p* = 0.73, *d* = 0.02; DASS-21: *t* = 0.43, *p* = 0.66, *d* = 0.03). There were also no significant differences in the ratio of individuals with NSSI behaviors between responders (35.3%, 85/241) and non-responders (30.4%, 110/362) at T2 (χ^2^ = 1.57, *p* = 0.20).

Baseline sociodemographic, self-injury behavior characteristics and clinical severity at T1 were summarized in Table 1. The average age of onset of NSSI was 14 years, and participants reported using an average of 4.71 NSSI methods (range 1–11). The most common NSSI methods were scratching (75%), biting (63%), pinching (55%), hitting (50%), wound picking, and cutting (45%). Compared with the Non-NSSI group, the NSSI group at baseline had a higher proportion of women, significantly poorer clinical mental health, and lower perceived social support.

**Table 1.** **Baseline sociodemographic and clinical characteristics pre-pandemic**.

		Non-NSSI	NSSI	Analysis		
		(N = 156)	(N = 85)	t-Student, Z, χ^2^	Effect size	*p*-value
Age M (SD)		21.50 (1.75)	21.26 (1.85)	–1.09	0.13	0.27
Sex (female), N (%)		125 (80.1)	77 (90.6)	4.43		0.03
Academic performance^1^, N (%)				3.07		0.21
	Good	119 (76.3)	56 (66.9)			
	Regular	31 (19.9)	25 (29.4)			
	Poor	6 (3.8)	4 (4.7)			
Psychological/psychiatric treatment^2^ (Yes), N (%)		71 (45.5)	56 (65.9)	9.15		0.002
BPQ Total, M (SD)		22.58 (12.88)	35.14 (13.37)	–6.42		<0.001
Past suicide attempt^3^, N (%)		3 (1.9)	17 (20)	23.62		<0.001
DERS-18, M (SD)		42.73 (12.69)	53.09 (14.85)	–5.15		<0.001
DASS-21, M (SD)		20.16 (13.98)	30.33 (15.43)	–4.88		<0.001
MSPSS, M (SD)						
	Family	20.85 (5.98)	17.18 (7.02)	–3.88		<0.001
	Friends	23.51 (4.66)	20.91 (5.68)	–3.49		<0.001
	Significant Other	23.01 (5.87)	21.29 (6.51)	–2.14		<0.03
	MSPSS Total	67.38 (12.60)	59.38 (15.13)	–4.07		<0.001
NSSI-features, M (SD)						
	NSSI-age of onset		14.13 (3.10)			
	Number NSSI-methods		4.71 (2.00)			
NSSI-function, M (SD)						
	Affect regulation		4.83 (2.08)			
	Interpersonal regulation		3.00 (1.94)			
	Positive feeling		2.24 (1.77)			

Note. Non-NSSI, group of young people without self-injury in the past year; NSSI, group of young people engaged in self-injury in the past year (≥5 days); ^1^ “*How would you define your current academic performance?*”; ^2^ “*In the last year, have you been to a psychologist or psychiatrist for mental health problems?*”; ^3^ “*Have you attempted suicide in the past?*”; BPQ Total, Borderline Personality Questionnaire; DERS-18, Brief Version of the Difficulties in Emotion Regulation Scale; DASS-21, Depression Anxiety Stress Scales; MSPSS, Multidimensional Scale of Perceived Social Support.

### Post-Pandemic Comparison

Table 2 depicts between-group differences (NSSI vs. Non-NSSI) at T2 in sociodemographic, COVID-19 related characteristics, academic performance, and clinical variables. Compared with the Non-NSSI group, participants in the NSSI group perceived a significantly higher impact of the COVID-19 pandemic on their academic performance and the degree to which the pandemic affected their mental health. However, there were no differences between groups regarding other COVID-19-related variables.

**Table 2.** **Differences between groups (NSSI vs. Non-NSSI) in sociodemographic, COVID-19 pandemic impact, academic performance, and clinical characteristics at T2**.

			GROUP TIME 1			
			Non-NSSI	NSSI	Analysis	
TIME 2			(N = 156)	(N = 85)	t-Student, Z, χ^2^	*p*-value
Age M (SD)			22.50 (1.55)	22.25 (1.85)	–1.09	0.27
Sex (female), N (%)			125 (80.1)	77 (90.6)	4.43	0.03
COVID-19 related Information (Yes), N (%)						
	*Participant*					
		Confirmed COVID Diagnosis	90 (57.7)	55 (64.7)	1.12	0.28
		Quarantine	108 (69.2)	65 (76.5)	1.42	0.23
		Hospitalization	16 (10.3)	9 (10.6)	0.007	0.93
	*Relatives (family members)*					
		Confirmed COVID Diagnosis	120 (76.9)	66 (77.6)	0.01	0.89
		ICU Hospitalization	25 (16)	11 (12.9)	0.41	0.52
		Death by COVID	25 (16)	6 (7.1)	3.94	0.047
Academic performance^1^			M (SD)	M (SD)		
	Your academic performance has worsened		2.45 (1.27)	2.88 (1.47)	–2.16	0.03
	Less motivated by the pandemic		2.53 (1.36)	3.01 (1.34)	–2.59	0.01
	Has been stressful to interact with your peers		2.81 (1.29)	3.19 (1.41)	–2.03	0.04
	Has been stressful to interact with your professors		2.83 (1.41)	3.09 (1.30)	–1.31	0.18
	To what degree has the pandemic affected your mental health? (range 1–10)		4.78 (2.37)	5.58 (2.63)	–2.45	0.01
Quality of life and healthy habits, M (SD)						
	Sleep (hours)		7.03 (0.96)	6.79 (0.98)	–1.77	0.07
	Daily meals (number)		3.20 (0.69)	3.10 (0.80)	–0.77	0.43
Alcohol and drug abuse, N (%)					2.95	0.39
	No		83 (53.2)	36 (42.4)		
	1–2 times × year		27 (17.3)	16 (18.8)		
	1–2 times × month		24 (15.4)	16 (18.8)		
	Weekly		22 (14.1)	17 (20)		
Psychological/psychiatric treatment^2^						
	Current treatment (during the last 12 months), N (%)		59 (37.8)	32 (37.6)	0.001	0.97
	Total number of months, M (SD)		6.75 (4.05)	8.06 (4.12)	–1.52	0.12

Note. Non-NSSI, group of young people without self-injury in the past year; NSSI, group of young people engaged in self-injury in the past year (≥5 days); ICU Hospitalization, intensive care unit; ^1^ If academic performance has worsened because of the pandemic; ^2^ “*In the last year, have you been to a psychologist or psychiatrist for mental health problems?*”.

### Changes in NSSI, Clinical, and Perceived Social Support After Pandemic

The temporal dynamics of NSSI from T1 to T2 showed a significant reduction in its prevalence (*p* < 0.001). Specifically, the percentage of participants engaging in repetitive NSSI (≥5 different days in the previous year) decreased from 35.3% (85/241) at T1 (January–February 2019/2020) to 8.7% (21/241) at T2 (June–July 2021/2022). Consequently, of the 85 participants who reported recurrent NSSI at T1, only 24.7% (n = 21) maintained this pattern at T2, while 75.3% (n = 64) no longer met the criterion for recurrent NSSI in the past year. Furthermore, only two participants in the total sample initiated NSSI *de novo* at T2 (without previous NSSI at T1).

Before running the LMMs, the normality assumption of the conditional residuals was assessed using the Shapiro-Wilk test and visual inspection of the respective Q-Q plots and histograms. The Shapiro-Wilk test was statistically significant for the residuals associated with BPQ Total (*W* = 0.99, *p* = 0.03), DASS-21 (*W* = 0.99, *p* = 0.002), and PSS (*W* = 0.97, *p* = 0.007). However, visual inspection of the Q-Q plots revealed no substantial deviations from the reference line across these variables, and the absolute values for skewness and kurtosis were all within the acceptable range of ±1. Given that LMMs are highly robust to minor violations of the normality assumption, particularly with a large sample size, we proceeded with the model fitting without data transformation.

**Fig. 1.**
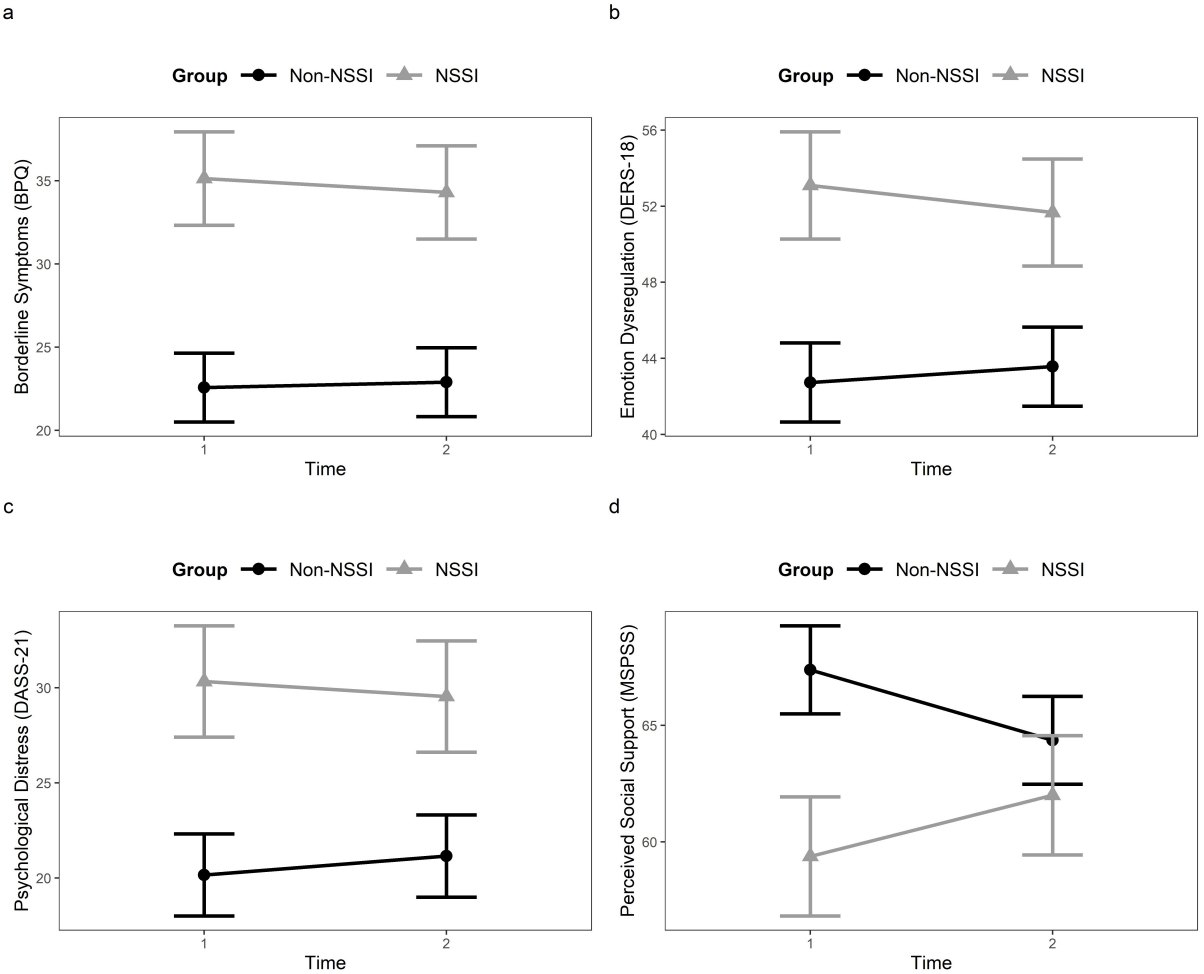
**Changes in clinical variables and perceived social support during the pandemic period in each group (NSSI vs Non-NSSI)**. Lines represent change from T1 to T2 in clinical measures (a–c) and perceived social support (d) by group.

Multilevel analyses explored differences between the groups in the trajectories of scores on clinical variables and perceived social support from time point T1 to T2 (see Table 3). We found a significant Group effect on all clinical variables (i.e., borderline traits, emotion regulation difficulties, and psychological distress), indicating that the NSSI group had worse outcomes at both T1 and T2 compared to the Non-NSSI group. Interestingly, both groups did not vary significantly in their clinical measures from T1 to T2, despite having experienced the pandemic (see Fig. 1a–c). Therefore, both the Non-NSSI and NSSI groups maintained their psychological status at follow-up.

**Table 3.** **Multilevel analysis of clinical variables and social support from Time 1 to Time 2 by NSSI-group**.

Multilevel Models			B	SE	*p*-value	M (SD)		M (SD)	
						NSSI group		Non-NSSI group	
						T1	T2	T1	T2
Clinical Measures									
	BPQ					35.14 (13.37)	34.31 (14.99)	22.58 (12.88)	22.90 (12.22)
		Time (T1–T2)	0.32	0.78	0.68				
		Group (NSSI vs. Non–NSSI)	12.56	1.77	<0.001				
		Time × Group	–1.15	1.32	0.38				
	DERS–18					53.09 (14.85)	51.67 (14.90)	42.73 (12.69)	43.56 (11.71)
		Time (T1–T2)	0.83	0.98	0.39				
		Group (NSSI vs. Non–NSSI)	10.36	1.78	<0.001				
		Time × Group	–2.25	1.65	0.17				
	DASS–Total					30.33 (15.43)	29.54 (13.71)	20.16 (13.98)	21.15 (12.41)
		Time (T1–T2)	0.99	1.09	0.05				
		Group (NSSI vs. Non–NSSI)	10.16	1.85	<0.001				
		Time × Group	–1.78	1.83	0.33				
Social Support Measures									
	PSS–Family					17.18 (7.02)	17.33 (6.89)	20.85 (5.98)	19.65 (6.89)
		Time (T1–T2)	–1.20	0.41	0.004				
		Group (NSSI vs. Non–NSSI)	–3.67	0.89	<0.001				
		Time × Group	1.35	0.70	0.05				
	PSS–Friends					20.91 (5.68)	22.38 (6.11)	23.51 (4.66)	22.79 (5.20)
		Time (T1–T2)	–0.71	0.41	0.08				
		Group (NSSI vs. Non–NSSI)	–2.60	0.71	<0.001				
		Time × Group	2.18	0.69	0.001				
	PSS–Significant					21.29 (6.51)	22.29 (6.67)	23.01 (5.87)	21.92 (7.28)
		Time (T1–T2)	–1.09	0.58	0.06				
		Group (NSSI vs. Non–NSSI)	–1.71	0.89	0.05				
		Time × Group	2.09	0.98	0.03				
	PSS–Total					59.38 (15.13)	62.00 (15.14)	67.38 (12.60)	64.36 (14.97)
		Time (T1–T2)	–3.01	1.00	0.002				
		Group (NSSI vs. Non–NSSI)	–8.00	1.92	<0.001				
		Time × Group	5.64	1.68	<0.001				

Note. NSSI group, young people engaged in self-injury in the past year (≥5 days); Non-NSSI, young people without self-injury in the past year; BPQ Total, Borderline Personality Questionnaire; DERS-18, Brief Version of the Difficulties in Emotion Regulation Scale; DASS-21, Depression Anxiety Stress Scales; PSS-Family, Perceived Social Support from family; PSS-Friends, Perceived Social Support from friends; PSS-Significant, Perceived Social Support from significant others; MSPSS, Multidimensional Scale of Perceived Social Support.

Subsequently, a significant Group effect was found on PSS, with the NSSI group reporting lower levels of social support compared to the Non-NSSI group. Moreover, a significant Group × Time interaction was observed for PSS, indicating that the temporal dynamics of PSS over time differed between the two groups (see Fig. 1d). Specifically, post-hoc analyses revealed a significant increase in perceived support from friends in the NSSI group from T1 to T2 (*t* = –2.03*, p* < 0.05), while the Non-NSSI group experienced significant decreases in perceived support from both family and friends (family: *t* = 3.96, *p* < 0.001; friends: *t* = 2.25, *p* < 0.05). These findings suggest that while the pandemic did not diminish PSS for individuals with NSSI, and even improved their perceived support from friends, it had a clear negative impact on PSS for individuals without NSSI (see Table 3 and Fig. 1d).

Given that the NSSI group experienced an unexpected increase in PSS, specifically from friends, alongside a decrease in NSSI behavior at T2, we next assessed whether this change in PSS predicted the observed remission of NSSI. We conducted a follow-up logistic regression to test whether PSS at T2 was associated with NSSI status at T2, controlling for PSS and NSSI status at T1. However, we found no evidence that the change in PSS from T1 to T2 was associated with the presence of NSSI at T2 (PSS Total: *OR* = 0.99 [95% *CI*: 0.96–1.04], *p* = 0.99; PSS Friends: *OR* = 0.99 [95% *CI*: 0.90–1.08], *p* = 0.84).

### Clinical Measures and Perceived Social Support at T1 predicting NSSI at T2

To explore whether clinical status and PSS at T1 were associated with the probability of maintaining or remitting NSSI behaviors at T2, multilevel logistic regression analyses were performed (Table 4). Univariate analyses revealed that borderline traits (BPQ), difficulties in emotion regulation (DERS), and psychological distress (DASS-21) significantly predicted a higher probability of presenting NSSI at T2. Conversely, PSS from family, friends, and significant others (MSPSS dimensions) predicted a lower probability of presenting NSSI at T2. However, when all significant variables were included in a multivariate model, only borderline traits and, marginally, psychological distress predicted a higher probability of presenting NSSI at T2 (see Table 4).

**Table 4.** **Multilevel logistic regression analyses to predict NSSI at Time 2**.

		Univariate analyses		Multivariate analyses	
		NSSI group (n = 21) vs. Non-NSSI group (n = 220) at TIME 2			
		OR (95% *CI*)	*p*-value	OR (95% *CI*)	*p*-value
TIME 1					
	Age	1.07 (0.84–1.37)	0.58		
BPQ		1.10 (1.06–1.14)	<0.001	1.11 (1.04–1.17)	<0.001
DERS-18		1.06 (1.02–1.09)	<0.001		
DASS-Total		1.07 (1.04–1.11)	<0.001	1.05 (1.00–1.10)	0.05
MSPSS					
	Family	0.93 (0.87–0.99)	0.02		
	Friends	0.88 (0.82–0.95)	<0.001		
	Significant others	0.92 (0.86–0.98)	0.009		

Note. The variable gender was not used as a predictor since the entire NSSI group at T2 was female. NSSI group, young people engaged in self-injury in Time 2; Non-NSSI group, young people without self-injury in Time 2; OR, Odds Ratio; BPQ, Borderline Personality Questionnaire; DERS-18, Difficulties in Emotion Regulation Scale; DASS-21, Depression Anxiety and Stress Scales; MSPSS, Multidimensional Scale of Perceived Social Support.

Subsequently, given the unexpected increase in PSS from T1 to T2 observed in the NSSI group, logistic regression analyses were used to explore whether PSS status at T2, controlling for PSS at T1, was associated with the probability of maintaining or remitting NSSI at T2 within the NSSI group. No evidence was found that the increase in PSS in the NSSI group was associated with a lower probability of NSSI at T2 (PSS-Total: *OR* = 0.99 [95% *CI*: 0.95–1.03]; PSS-Family: *OR* = 0.95 [95% *CI*: 0.86–1.04]; PSS-Friends: *OR* = 0.98 [95% *CI*: 0.89–1.08]; PSS-Significant Other: *OR* = 1.01 [95% *CI*: 0.93–1.09]).

## Discussion

This longitudinal study, initiated immediately before the COVID-19 pandemic, provided a unique opportunity to examine whether young individuals with NSSI experienced the pandemic differently from their peers without NSSI. Our findings revealed a striking divergence in the long-term impact of the pandemic on these groups. Specifically, while individuals with NSSI exhibited greater vulnerability in psychological and academic outcomes, they unexpectedly showed significant improvements in PSS and a reduction in NSSI behaviors during the pandemic. These results underscore that the pandemic’s impact was heterogeneous among young people and highlight the crucial role of social support in managing NSSI, especially in vulnerable populations. Notably, youth who engage in NSSI behaviors, often characterized by poorer psychopathological indicators, did not necessarily experience a decrease in perceived social support. This contrasts with young people without NSSI behaviors and with lower psychopathological risk, who did experience a negative impact on their perception of social support. These findings highlight promising new avenues for interventions that strengthen social support networks, aiming to prevent and reduce NSSI in young people.

Contrary to expectations, our results indicate that the long-term impact of the pandemic on self-injury behaviors was less detrimental than anticipated. Descriptively, the prevalence of NSSI decreased from 35% at baseline to 8.7% at follow-up. This unexpected finding contrasts with many previous studies and meta-analyses reporting a negative effect of the pandemic on self-injury and suicide [20, 21, 33]. For instance, the lifetime prevalence of NSSI among Swiss adolescents was approximately 17% before the pandemic and increased to 27.6% during the 2020–2021 pandemic period [34]. Another study found that pandemic-related perceived stress was linked to an increase in NSSI among youth. However, this effect was only significant for those who reported negative parenting, while positive parenting had a buffering effect against NSSI [35]. Furthermore, the pandemic appears to have affected youth differentially based on both age group and the type of self-injurious thought or behavior. Specifically, higher rates of NSSI were observed among late adolescents compared to young adults [36], and the pandemic seems to have exerted a greater incremental effect on suicidal behaviors in comparison with NSSI [20]. Finally, other studies found a similar NSSI prevalence before and after the pandemic [25, 37].

Our results are aligned with a previous study that explored the lived experience of NSSI during the pandemic in a similar sample of young people. In that study, 80% of respondents reported no change in the urge or behavior of NSSI due to the pandemic, with a perceived reduction in social stress and more time for self-care as protective factors. Conversely, only 20% reported being negatively affected by isolation and additional pandemic-related stressors, which influenced their desire to self-harm [38]. These findings support the hypothesis that individuals with NSSI may have either faced fewer unpleasant interpersonal situations or developed new coping mechanisms during the pandemic. For instance, not being exposed to academic stressors and difficult interpersonal dynamics may have led to a decrease in the NSSI urge. Alternatively, young adults who engaged in self-injury at T1 may have learned to manage their emotions more adaptively two years later, or they may have shifted from NSSI to other types of maladaptive behaviors, such as substance use [39]. Finally, our results may also reflect the natural trajectory of NSSI, which typically declines in late adolescence and young adulthood [5].

A divergence in social support trends between participants with and without a history of NSSI was found. Previous research has consistently reported poor PSS as a consequence of pandemic social restriction policies [9, 40]. While we observed this decline in non-NSSI participants, it was not seen in the NSSI group. Interestingly, participants with NSSI at baseline, who initially reported lower PSS compared to their non-NSSI counterparts, maintained or slightly increased their PSS at follow-up, regardless of their NSSI status at T2. This paradoxical result is significant given that PSS is a critical protective factor against NSSI and broader psychopathology [9, 12]. On one hand, participants without NSSI experienced an expected long-term reduction in PSS as a significant reduction in their positive social interactions. Previous evidence shows that social support was adversely affected by the COVID-19 pandemic and this impacts on quality of life, depression, and mental health outcomes [9, 10, 41]. On the other hand, for participants with NSSI the isolation may have resulted in the avoidance of social environments that typically trigger NSSI, thus limiting face-to-face interactions and possibly reducing the incidence of NSSI that we also found (as mentioned above). These changes in social interactions may have persisted over time, enabling our long-term study to capture them even though social restrictions were no longer in place at the time of the T2 assessment [9, 12].

Participants with NSSI showed poor mental health status and rated their academic performance as the poorest after the pandemic. This group reported less motivation in studies and worse academic performance, highlighting their higher vulnerability to stressors affecting their main activities. While we hypothesized that the long-term consequences of the pandemic on NSSI individuals would result in clinical worsening, we did not find significant differences between pre- and post-pandemic assessment. However, this finding should be interpreted with caution, as these individuals already exhibited significant clinical severity at baseline. The lack of statistical differences does not detract from the fact that they continue to score very low, indicating persistent high levels of suffering despite having ceased NSSI. Additionally, it is possible that they were unable to access mental health services during the pandemic, which may have contributed to their lack of improvement. In this regard, previous studies have suggested that NSSI may be considered as an early indicator of psychological vulnerability [42, 43, 44]. Our findings support this approach, as NSSI at baseline (versus lack of NSSI) suggests poor long-term mental health outcomes. The moderate long-term impact on mental health observed in participants without NSSI further supports this notion. Finally, participants who continued to engage in NSSI at T2 were associated primarily with borderline psychopathology, rather than with emotional dysregulation, psychological distress, or dimensions of social support, when all of these predictors were controlled in a multivariate analysis. This result suggests that, although social support plays a role in NSSI, the presence of borderline features may be a more critical factor in maintaining NSSI behaviors over time [12].

Finally, these findings carry several clinical implications. Even with the observed decline in NSSI frequency over time in young adults, the long-term vulnerability to mental health problems underscores the need for early interventions, especially following any new NSSI episodes. These interventions can be implemented and adapted within university settings, particularly targeting first-year students who report NSSI and other psychological difficulties. This specific group could benefit from personalized interventions focused on developing social adaptation skills, emotional regulation, and interpersonal effectiveness to manage academic stress and reduce high-risk behaviors [45].

The present study has several limitations. First, only 40% of the overall sample completed both assessments and, although there were no significant differences between responders and non-responders, the estimates of NSSI frequency may be biased. For instance, participants with greater psychological distress or more severe NSSI may have experienced differential attrition by T2. Furthermore, several factors likely contributed to this low retention rate: the long interval between the T1 and T2 assessments, the lack of financial compensation at T2, and the reliance on email as the sole means of contact, which may have negatively affected participants’ receipt of information and motivation to continue their participation. Second, the 2.5-year follow-up period may be too long, as the acute pandemic and quarantine mainly occurred in 2020–2022, and its effects may have mitigated by mild-2022. However, this follow-up design was conceptualized before the pandemic, providing an opportunity to investigate long-term effects in this population. Third, only self-report measures by mail were used, potentially introducing recall bias. For instance, the number of days of NSSI reported in the past year is vulnerable to imprecision, potentially resulting in either underestimation or overestimation. Fourth, other significant variables such as mental health comorbidities were not explored through clinical interviews. Fifth, the sample was largely skewed toward females, consistent with previous studies showing females are more likely to report a history of NSSI than males [46]. Finally, the lack of an objective measure of social interactions at baseline limit our conclusions.

## Conclusion

This longitudinal study shows that the COVID-19 pandemic had a long-term divergent impact on young adults with and without a history of NSSI. While individuals without NSSI showed an expected decline in psychological well-being and PSS, individuals with NSSI exhibited a stable level of psychological distress, PSS and, importantly, a significant decline in NSSI frequency. These findings suggest that individuals with a history of NSSI should be considered a particularly vulnerable population within university settings. Despite a significant reduction in the frequency of NSSI over time, these individuals maintained elevated levels of psychopathology and psychological distress. The results of this study underscore the need to support young people with a history of NSSI within higher education, emphasizing the importance of social support dynamics during their academic journey.

## Availability of Data and Materials

Data related to this study can be obtained from the corresponding author upon reasonable request.
